# Low-pressure exposure influences the development of HAPE

**DOI:** 10.1515/biol-2022-1029

**Published:** 2025-04-01

**Authors:** Bo Wang, JinXiu Hou, Jing Li, Shuaijie Pei, Keliang Xie, Wenbo Liu

**Affiliations:** Laboratory of Anesthesia and Critical Care Medicine in Colleges and Universities of Shandong Province, School of Anesthesiology, Shandong Second Medical University, Weifang, Shandong, China; Department of Gastroenterology, Weifang People’s Hospital, Weifang, Shandong, China; Department of Critical Care Medicine, Tianjin Medical University General Hospital, No. 154, Anshan Road, Heping District, Tianjin, China; Department of Anesthesiology, Tianjin Medical University General Hospital, Tianjin, China; Central Laboratory of the First Affiliated Hospital, Shandong Second Medical University, No. 151, Guangwen Street, Kuiwen District, Weifang, Shandong, China

**Keywords:** high-altitude pulmonary edema, hypobaric hypoxia, inflammation, mountain sickness

## Abstract

High-altitude pulmonary edema (HAPE) is non-cardiogenic pulmonary edema that occurs in healthy individuals who are unable to acclimatize when they ascend above 2,500–3,000 m above sea level in a few days. The pathogenesis of HAPE is complex and it is difficult to explain it by a single mechanism. Currently recognized pathogenesis is related to altered pulmonary hemodynamics, increased pulmonary capillary permeability, inflammatory response, dysregulated sodium–water transport, and impaired alveolar fluid clearance, and it is caused by a combination of causes. A high-altitude environment is both hypobaric and hypoxic, and whether hypobaria contributes to the pathogenesis of HAPE is unknown. We established a model in which 30 Institute of Cancer Research Mice (ICR mice) were divided into normal control (C, *n* = 6), normobaric hypoxic (CH, *n* = 8), hypobaric normoxic (HC, *n* = 8), and hypobaric hypoxic (HH, *n* = 8) groups. The simulated altitude was 5,000 m above sea level. Lung tissue wet/dry (W/D) ratios were determined, and molecular and histologic examinations were performed on each animal. W/D ratios were increased in the CH, HC, and HH groups compared to controls. ELISA revealed increased IL-1β expression in the HC and HH groups and markedly elevated TNF-α levels in the HH group. Histologic sections disclosed septal thickening and edema with inflammatory cell infiltration in the CH, HC, and HH groups compared with the control group. The HH group exhibited the most severe histopathologic abnormalities that included septal rupture. Electron microscopy demonstrated injury to endothelial and epithelial membranes and disruption of the integrity of the air–blood barrier after hypobaric and/or hypoxic exposures. Our study establishes a direct link between the histologic and physiologic findings of HAPE-like disease and demonstrates that normoxic hypobaria incites HAPE-like pathology. Furthermore, hypoxia and hypobaria act synergistically and stimulate inflammation in ICR mice. Therefore, we suggest that hypobaria plays an important role in the pathogenesis of HAPE.

## 1 Background

High altitude pulmonary edema (HAPE) is a form of non-cardiogenic pulmonary edema that manifests when individuals ascend to altitudes exceeding 2,500–3,000 m above sea level within a short time period [1,2,3,4]. This disorder is distinguished by aberrant lung function and complications that may be fatal in severe cases. Prompt reduction of altitude or timely intervention can mitigate disease severity. Due to the flourishing tourism industry in high-altitude locations and the imperative for social advancement and military operations, an increasing number of individuals residing at low-altitude access locations above 2,500 m elevation rapidly through vehicles such as aircraft. Consequently, a minority of individuals may experience HAPE soon after arrival owing to inadequate acclimatization. Consequently, HAPE has emerged as an issue that cannot be disregarded.

The pathogenesis of HAPE has garnered significant attention since its initial report. Currently, it is widely accepted that hypoxia induces pulmonary hypertension. Hypoxic pulmonary vasoconstriction (HPV) is one of the key mechanisms in the development of HAPE. During acute hypoxia, pulmonary vasoconstriction, called HPV, can occur, and severe HPV leads to excessive increases in pulmonary artery pressure. Due to the uneven degree of vasoconstriction in the lungs, pulmonary blood flow is forced to the less constricted vessels, and the pulmonary capillary bed becomes overfilled with fluid, resulting in “stress failure” and fluid leakage into the interstitium and alveoli, leading to the development of HAPE [5,6,7,8,9]. It disrupts water and sodium transport in lung epithelium and reduces expression of related proteins: hypoxia may lead to reduced expression of proteins responsible for sodium and water transport on the alveolar epithelium, such as Na^+^-K^+^-ATPase. Reduction of these proteins leads to decreased sodium and water transport, making it impossible to remove fluid from the alveoli in a timely manner. Imbalance of sodium and water transport: Sodium and water transport within the alveoli may become imbalanced due to decreased expression and/or dysfunction of the relevant proteins. This can lead to the accumulation of fluid in the alveoli, which in turn can lead to the development of pulmonary edema. An imbalance in sodium–water transport leads to fluid accumulation in the alveoli, further aggravating the degree of pulmonary edema. This affects the ventilation and air exchange function of the lungs, leading to worsening symptoms such as dyspnea and hypoxemia. Sodium–water transport imbalances may also affect the effectiveness of HAPE treatment. For example, when using medications such as diuretics, the efficacy of the medication may be limited if the sodium–water transport system is not effectively restored [10] and alters pulmonary vascular permeability, among other factors. Hypoxia is the underlying cause of the development of HAPE. When the organism is at high altitude, pulmonary vasoconstriction occurs due to the scarcity of oxygen in order to increase the resistance to blood flow, which raises pulmonary arterial pressure. This elevated pressure may lead to damage to the endothelial cells of the pulmonary capillaries, which in turn increases vascular permeability. Hypoxia may also trigger an inflammatory response, leading to inflammatory cell infiltration and the release of inflammatory mediators in the walls of the pulmonary vasculature. These inflammatory mediators can further damage pulmonary vascular endothelial cells and increase vascular permeability.

Under hypoxic conditions, sympathetic nerves become excited and release neurotransmitters such as catecholamines. These neurotransmitters may affect pulmonary vascular permeability by regulating vascular smooth muscle contraction and diastole [11,12]. Physiologic stressors in the high-altitude environment include both hypoxia and hypobaria; whether normoxic hypobaria causes HAPE is unclear. In this study, we hypothesized that hypobaric exposures may cause lung injury and, thus, HAPE. To test this hypothesis, we developed a model in which mice were subjected to hypobaric exposures. We assessed wet/dry (W/D) ratios, histology, the ultra-microstructure of lung tissue, and the expression levels of the tight junction protein claudin-5 and pro-inflammatory cytokines. To the best of our knowledge, this model offers the first demonstration of a direct link between hypobaric exposure, tissue injury, and abnormal cytokine expression.

## 2 Methods

### 2.1 Animals

Healthy male 8-week-old ICR mice (Jinan Pengyue Animal Breeding Co., Ltd [China] and utilized under animal production license [No. SCXK (Lu)20220006]) were used for this study. Prior to the start of the experiment, the mice were exposed to a 12-h diurnal light cycle for 2 days to acclimatize them to the experimental environment [13]. All animal experimental protocols were approved by the Ethics Committee and were conducted in accordance with the Guidelines for the Use and Care of Research Animals published by the US National Institutes of Health [14].


**Ethical approval:** The research related to animal use has been complied with all the relevant national regulations and institutional policies for the care and use of animals.

### 2.2 Experimental design

Mice were placed in a hypobaric decompression chamber to simulate a high-altitude environment. The partial pressure of oxygen in inhaled air was calculated as PIO_2_ = (PB–P_H_2_O_) × FIO_2_. Hypoxia was defined as a partial pressure of oxygen that was lower than the partial pressure of oxygen at 1 standard atmospheric pressure. If the ambient pressure is halved, FIO_2_ must be raised to 0.40 to maintain the partial pressure of oxygen in inhaled air. The mice were divided into four groups based on their exposures, and comprised normal control (101 kPa, FIO_2_ 0.21, C), normobaric hypoxic (101 kPa, FIO_2_ 0.10, CH), hypobaric normoxic (50 kPa, FIO_2_ 0.40, HC), and hypobaric hypoxic (50 kPa, FIO_2_ 0.10, HH) groups. After 7 days at 5,500 m, animals were subjected to the treatment.

### 2.3 Lung W/D ratio

The mice were placed in the supine position following anesthesia induced by intraperitoneal injection of 1% pentobarbital sodium, and a cannula was inserted into the trachea after tracheotomy. Fresh lung tissue was obtained from mice. After thoracotomy, the lungs were isolated. Exsanguinated entire lungs were weighed on an electrical scale and then dried in an oven at 55°C for 72 h before the recording of dry weight [15]. Lung W/D ratios were calculated as previously described.

### 2.4 Lung histopathology

Right lung tissue samples were stored in 4% paraformaldehyde for 48–72 h for light microscopy or in 2.5% glutaraldehyde for 48–72 h for electron microscopy [16]. Lung tissue for light microscopy was embedded in paraffin, sectioned, and stained with hematoxylin and eosin [15]. Lung tissue for electron microscopy was fixed in 1% lanthanum nitrate, rinsed twice with 0.1 M sodium dimethyl arsenate buffer, and postfixed with 1% osmium tetroxide for 2 h. The specimens were rinsed twice with 0.1 mol/L sodium dimethyl arsenate, dehydrated, sectioned, contrasted with uranyl acetate and lead citrate, and viewed by transmission electron microscopy [15].

### 2.5 Molecular biology analysis

Fresh lung tissue was obtained from mice after anesthesia. Blood was flushed with PBS pre-cooled at 4°C, and the remaining PBS in the tissue was absorbed with filter paper. Then, 30 mg tissue was weighed, and 200 µL of RIPA lysate and 2 µL of PMSF were added. After the tissue fragments were fully ground, they were cleaved at 4°C for 30 min and then centrifuged at 12,000 rpm to extract the supernatant and to obtain the histone extract. The BCA protein assay kit was used to measure protein concentrations. Protein loading was set at 30 µg/well. In short, the protein sample was mixed with 5× loading buffer, loaded into SDS-PAGE gel, and transferred to a PVDF membrane. The PVDF membrane was sealed with 5% BSA sealing solution for 4 h and subjected to five TBST washings of 5 min duration. Claudin-5 was detected using an affinity-purified mouse monoclonal antibody (1:1,000) and horseradish peroxidase-conjugated goat anti-rabbit IgG by western blotting. To confirm the equivalent loading of samples, the same membrane was incubated with anti-beta actin. For each 20 mg of lung tissue, we added 200 µL of 1 × PBS pre-cooled at 4°C. The sample was then fully ground on ice and centrifuged at 4°C at 12,000 rpm for 10 min to obtain the supernatant. IL-1β and TNF-α were detected by ELISA.

### 2.6 Statistical analysis

SPSS version 26.0 software (IBM SPSS Statistics, IBM Corporation) was used for statistical analysis. Quantitative data were expressed as a percentage, and measurement data as mean ± SD. Comparisons between multiple groups were performed using ANOVA and Bonferroni tests [14,17]. A *P* < 0.05 between groups was considered statistically significant. The Pearson test was performed to assess data correlation, and *r* is the correlation coefficient (−1, *r*, 1). Statistical comparisons were always made with the control group (C) unless otherwise indicated.

## 3 Results

### 3.1 Lung W/D ratio

**Figure 1:**
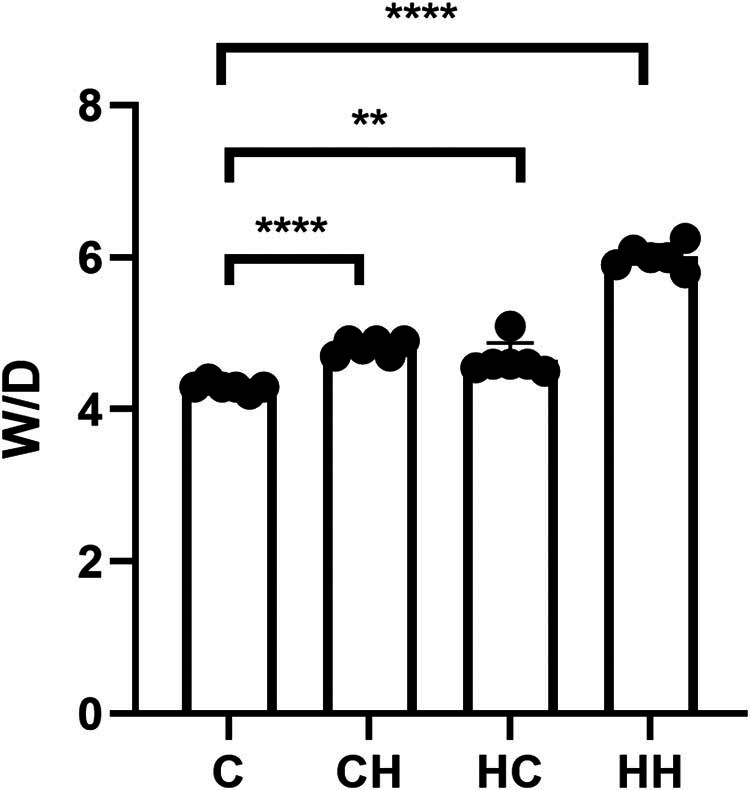
Statistical histograms of W/D ratios. W/D ratios were significantly higher in the CH (*P* < 0.0001), HC (*P* < 0.01), and HH (*P* < 0.0001) groups compared to the control (C) group. Statistical results are expressed as the mean ± SD.

The pulmonary W/D ratio indicates the severity of pulmonary edema [18]. Compared to the control group, lung W/D ratios were higher in the CH and HC groups and highest in the HH group. The W/D ratios of the CH and HC groups were similar (Figure 1).

### 3.2 Inflammation

**Figure 2:**
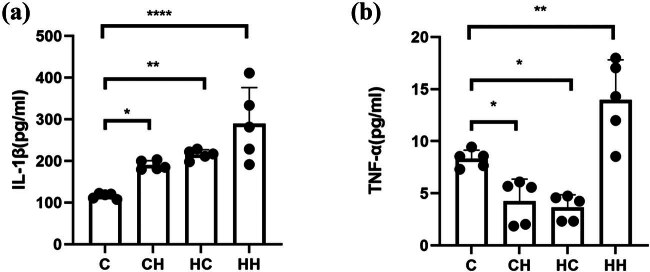
Statistical histograms of (a) IL-1β and (b) TNF-α expression. Compared with the control (C) group, mean IL-1β levels were significantly elevated in the HH (****P < 0.001) and HC (**P < 0.01) groups but not significantly changed in the CH group (***P < 0.001). TNF-α expression decreased in the HC and CH groups (*P* < 0.05) and increased significantly in the HH group (*P* < 0.01).

Hypobaric hypoxic exposures can induce inflammation [19]. ELISA of lung tissue homogenate showed that compared with the control group, mean IL-1β levels were significantly elevated in the HH and HC groups but not significantly changed in the CH group (Figure 2a). Surprisingly, TNF-α expression showed a decreasing trend in the HC and CH groups but was significantly elevated in the HH group (Figure 2b). The underlying mechanisms deserve further exploration.

### 3.3 Detection of tight junction protein claudin-5

**Figure 3:**
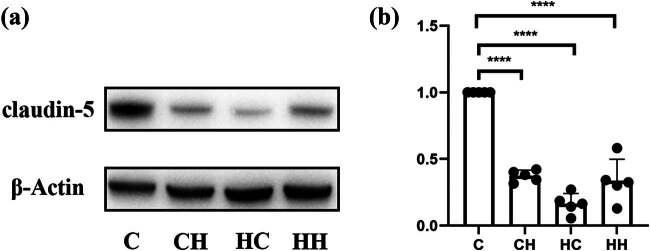
Claudin-5 expression. (a) Western blots of claudin-5 in the four groups. (b) Statistical histogram of Western blot results for claudin-5. Compared with controls, claudin-5 expression was significantly reduced in the other three groups (*P*＜0.0001). Full-length blots/gels are presented in Supplementary Figure 1.

Claudin-5 is the main structural determinant of the paracellular endothelial barrier [20]. Its expression promotes the closure of tight junctions, which reduces vascular permeability and thus enhances the barrier function of endothelial cells [21,22,23]. To confirm whether the hypobaric environment affects claudine-5 expression, we compared the expression of claudin-5 under different treatment conditions. Compared with the control group, both hypobaric and hypoxic exposures reduced the expression of claudin-5 (Figure 3).

### 3.4 Light microscopy

**Figure 4:**
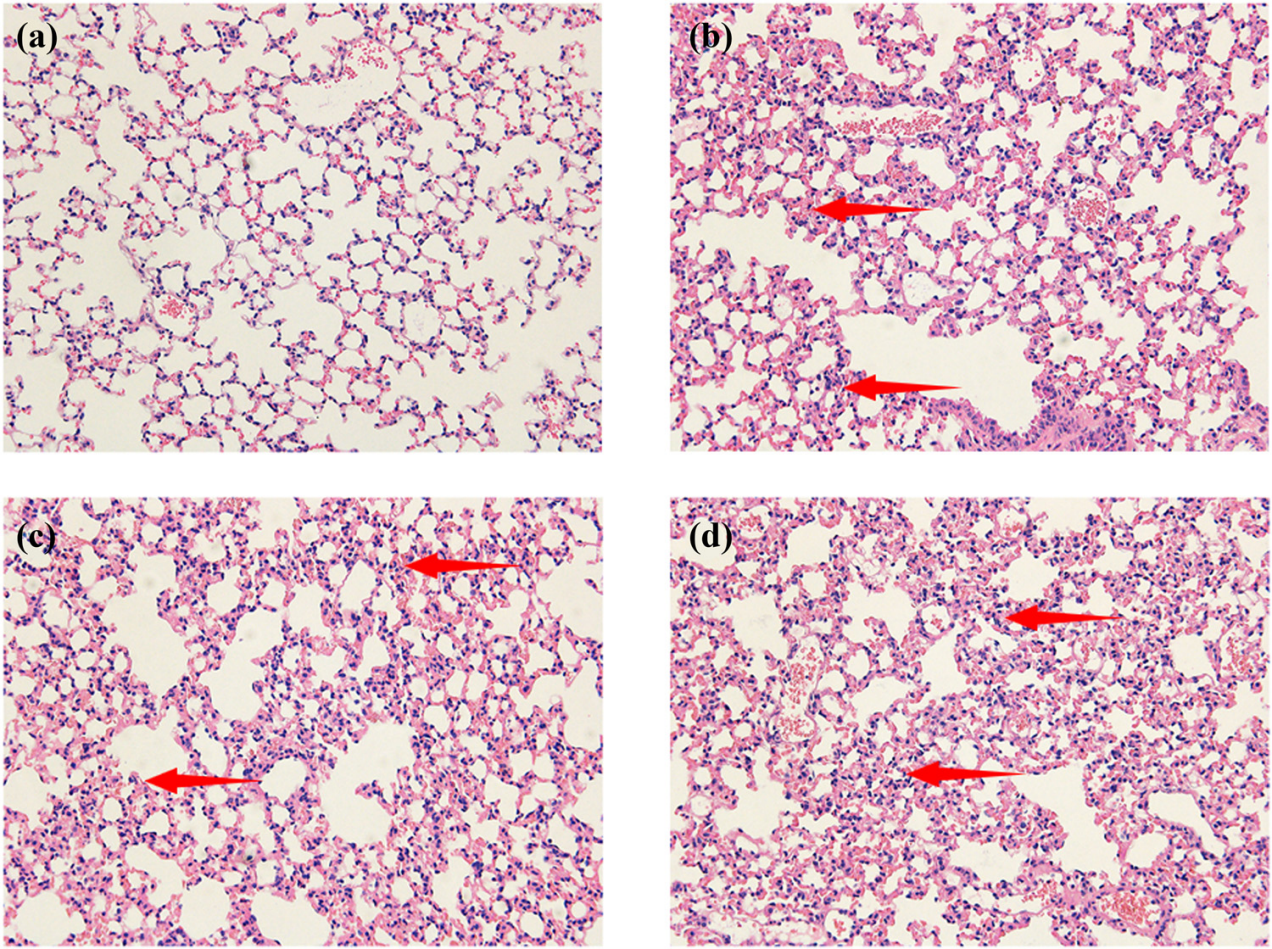
Lung histopathology (hematoxylin and eosin staining). (a) Normobaric control group. The picture shows the normal alveolar structure, with no edema fluid or protein leakage. (b) Normobaric hypoxic group. Thickening of the pulmonary septa with disruption of integrity, presence of congestion in the pulmonary vasculature, and presence of infiltrating inflammatory cells. (c) Hypobaric normoxic group. Varying degrees of interstitial thickening, the presence of congestion in the pulmonary vasculature, and the presence of infiltrating inflammatory cells. (d) Hypobaric hypoxic group. The destruction of the alveolar structure is the most severe. The pulmonary septa are markedly thickened, and pulmonary vascular congestion is evident. Thickening and breakdown of the pulmonary septa as indicated by the arrows.

Normobaric normoxic controls exhibited normal alveolar structure with no proteinaceous transudates. The lung tissues of CH and HC animals were dense, with signs of pulmonary vascular congestion, alveolar hemorrhage, septal thickening, and intraluminal proteinaceous transudates. Lung injury was most severe in the HH group (Figure 4).

### 3.5 Electron microscopy

**Figure 5:**
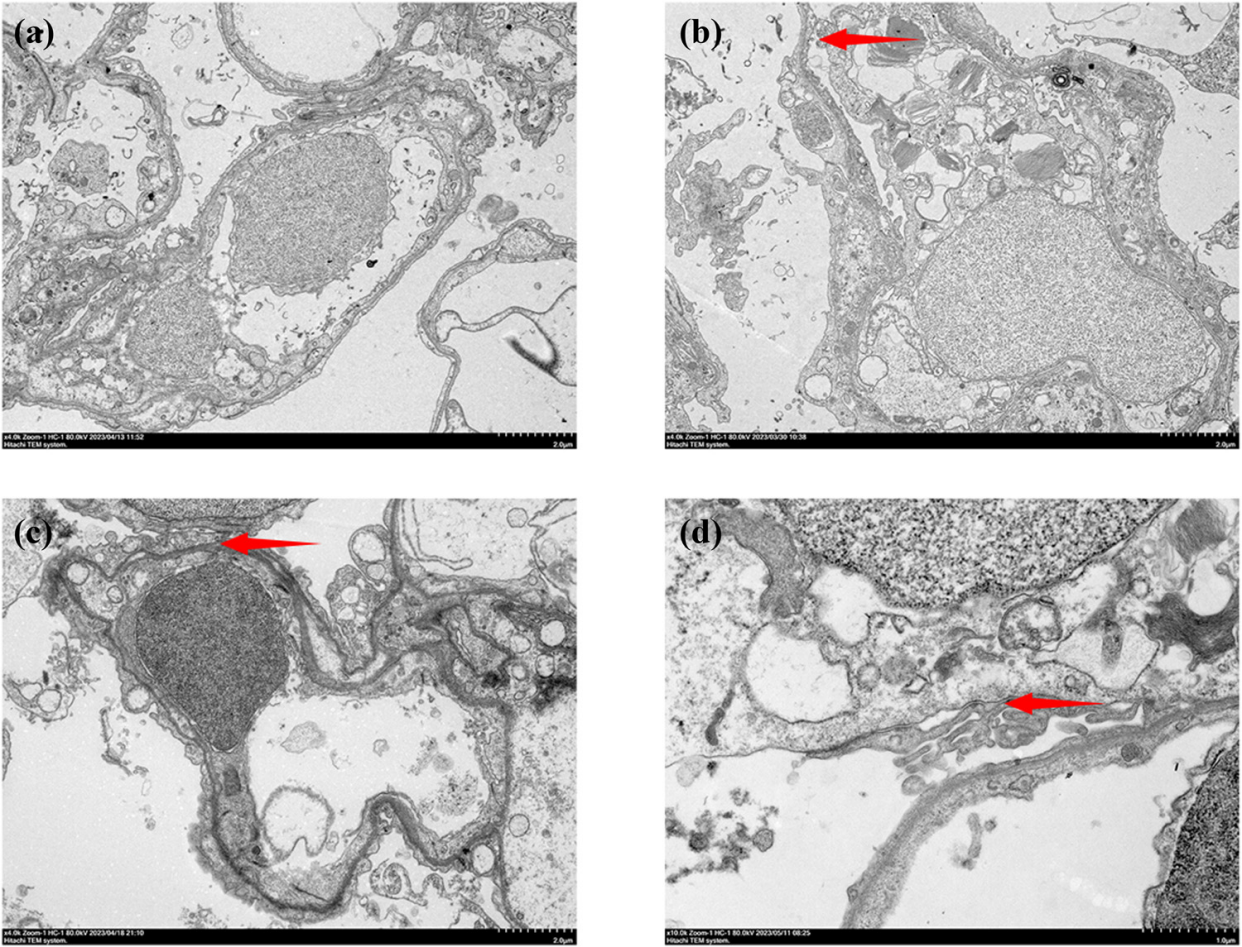
Ultrastructure of lung tissue. (a) Normobaric control group. (b) Normobaric hypoxic group. (c) Hypobaric normoxic group. (d) Hypobaric hypoxia group. Arrows indicate edema of the endothelial layer and epithelial lining.

Images from the control group disclosed normal ultrastructural appearance of epithelial and endothelial barriers (Figure 5a). In contrast, profound changes in the lung ultrastructure were observed after hypobaric and hypoxic exposures, characterized by disruption of the alveolar epithelium and capillary endothelium, as well as discontinuities in the blood–gas barrier. The bilayer membrane structure of the capillary wall disintegrated, with an ill-defined or beaded appearance (Figure 5b and c). Other findings included edema of the endothelial layer and epithelial lining and RBCs in capillaries and alveoli and complete rupture of the blood–gas barrier, including epithelium, endothelium, and basement membrane. The integrity of the air–blood barrier was lost in the HH group (Figure 5d).

## 4 Discussion

Endothelial barrier function is regulated by transcellular and paracellular pathways. The transcellular pathway translocates large molecules via active transport; the paracellular pathway is regulated by endothelial intercellular junctions, allowing small molecules to transit in various forms [24,25]. Endothelial intercellular junctions consist of a combination of tight, adherents, and gap junctions (tight junction [TJ], adherens junction, and gap junction, respectively), with TJs dominating the endothelial barrier function [26,27,28]. TJs are transmembrane protein complexes composed of claudin family proteins in combination with a variety of linker proteins, which are connected to intracellular TJ-related proteins and the cytoskeleton for signaling and stabilizing cellular structure, as well as being subjected to modification and regulation [29]. Among the TJ protein families, the claudin family includes the most members that exhibit a certain degree of tissue specificity. Their unique four-fold transmembrane structure plays an important role in endothelial barrier function. Upregulation of claudin-5 expression can maintain the integrity of TJs, stabilize the structure of endothelial cells, and reduce endothelial permeability [30]. Kakogiannos et al. associated downregulation of claudin-5 expression in the pulmonary and cerebral vasculature of JAM-A-deficient mice with significantly increased vascular permeability. Furthermore, the upregulation of claudin-5 expression promoted the closure of the transporter pathway in TJs and enhanced vascular endothelial function [31]. It has been shown that hypoxia-induced endothelial barrier dysfunction and HPC increased claudin-5 expression, reduced cellular damage, and maintained the integrity of the endothelial barrier [32], which agrees with our findings. Additionally, in a study on the blood–brain barrier, it was found that claudin-5 expression likewise decreased after the blood–brain barrier was disrupted, causing edema [33]; this finding likewise suggests an important role for claudin-5 in the endothelial barrier function. A study by Sanwal et al. found that increased claudin-5 expression improved the endothelial barrier function in patients with lung injury [34], and this finding provides new ideas for the subsequent prevention as well as treatment of HAPE and warrants further study. In the present study, we found that claudin-5 expression decreased after hypobaric and hypoxic exposures. These results indicate that exposure to hypobaric and hypoxic environments significantly reduced claudin-5 expression at the protein level, impaired pulmonary vascular barrier function, and increased vascular permeability.

Another factor thought to promote the pathogenesis of HAPE is inflammation [19,35]. Macrophages are the most prominent inflammatory cell type in the alveolar and pulmonary vessel walls of HAPE patients. In addition, inflammatory cells such as neutrophils are also involved in the pathogenesis of HAPE. Under severe hypoxic conditions, vascular endothelial cells and circulating myeloid cells release pro-inflammatory cytokines such as IL-1β, IL-6, IL-8, and TNF-α. The release of these inflammatory factors is quite rapid and occurs within hours, and thus may be an early event in HAPE. Hypoxia also alters the expression of various vasoactive and inflammatory factors in pulmonary nonvascular cells, such as macrophages, lymphocytes, and dendritic cells. Recent studies have shown that the expression of inflammation-related genes (e.g., MMP8, MMP9, IL-17β, and Timp1) is upregulated in the lungs of rats exposed to the same hypoxic conditions. Under hypoxic conditions, macrophages undergo recruitment and activation in the lungs, releasing inflammatory factors and vasoconstrictive substances, which are involved in promoting the process of increased hypoxic pulmonary arterial pressure, further contributing to the development of HAPE. A range of inflammatory mediators are involved. Hypoxia-induced rupture of the pulmonary vascular endothelium, exposure of the basement membrane, and the release of antigens and reactive oxygen species (ROS) can trigger inflammation. Hypoxic stress and blockade of electron transport in the mitochondrial respiratory chain can lead to increased ROS levels. Mitochondrial complex III is a major source of ROS under hypoxic conditions [36,37,38]. IL-1β, secreted as a result of ROS activation of NLRP 3 inflammasomes, is a major pro-inflammatory mediator of lung injury [39]. Our finding that pulmonary IL-1β expression was increased in the HC and HH but not in the CH group suggests that hypobaria, rather than hypoxia, was the major stimulus of IL-1β release. TNF exerts tumor-suppressive effects *in vivo* and induces cytotoxicity in PMNs and T effector cells, which kill tumor cells, virus-infected cells, and other cells and inhibit viral replication [40,41,42]. Low-intensity noxae (hypobaria or hypoxia) reduce the protective effect of TNF and inhibit TNF expression, as we observed in the CH and HC groups. However, when the animals were subjected to a combined hypobaric and hypoxic challenge (HH group), TNF expression increased, showing a pro-inflammatory effect. Moreover, hypobaria and hypoxia promoted the inflammatory response of HAPE synergistically. Our *in vivo* findings affirm that inflammation plays an important role in the pathogenesis of HAPE [19] and suggests that HAPE may be stimulated by hypobaria in the absence of hypoxia.

Furthermore, electron microscopy showed that either hypoxia or hypobaria alone caused only slight alveolar epithelial and capillary endothelial edema/rupture, whereas their combination exacerbated these ultrastructural abnormalities. The disruption of alveolar capillary membranes by combined hypoxia and hypobaria was confirmed by a higher W/D ratio in HH mice. Epithelial and endothelial pathology was also confirmed by light microscopy.

## 5 Limitation

The experimental environment may not fully simulate the real environment, resulting in discrepancies between the experimental results and the actual situation. The experimental design may be limited by the current theoretical framework, resulting in experiments that do not fully explore all possible influences. Experiments on pulmonary vascular pressure and cardiac changes are missing to further test the hypothesis, and we will refine the experiments in future studies.

## 6 Conclusions

This study showed that hypobaria in a setting of normoxia can trigger the pathogenesis of HAPE. Our murine model demonstrated that hypobaric exposure caused histopathologic abnormalities in lung tissue and incited inflammation. In addition, hypobaria inhibited the expression of the tight junction protein claudin-5, thereby disrupting the integrity of the air–blood barrier. Although our model is not representative of all cases of HAPE, these results suggest that hypobaric exposure could represent a new therapeutic entry point for the prevention and treatment of HAPE.

## Supplementary Material

Supplementary Figure
