# Supplementary figures and images for: Low-pressure exposure influences the development of HAPE
Source: Open Life Sci. 2025 Apr 1;20(1):20221029. doi: 10.1515/biol-2022-1029 (PMC11964184; doi:10.1515/biol-2022-1029)

## Supplementary material

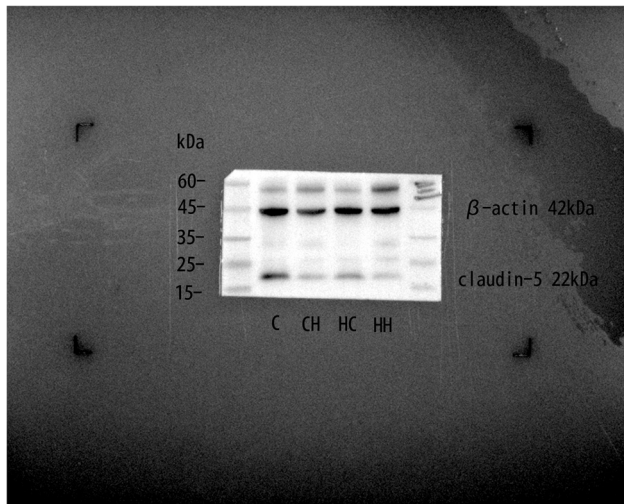

**Figure S1:** Full-length blots/gels for claudin-5.

Supplement: Supplementary Figure [file biol-2022-1029-sm.pdf]
